# Additional calcar support using a blade device reduces secondary varus displacement following reconstruction of the proximal humerus: a prospective study

**DOI:** 10.1186/s40001-015-0178-5

**Published:** 2015-10-07

**Authors:** Marc Beirer, Moritz Crönlein, Arne J. Venjakob, Tim Saier, Marcus Schmitt-Sody, Stefan Huber-Wagner, Peter Biberthaler, Chlodwig Kirchhoff

**Affiliations:** Department of Trauma Surgery, Klinikum rechts der Isar, Technical University of Munich, Ismaningerstrasse 22, 81675 Munich, Germany; Berufsgenossenschaftliche Unfallklinik Murnau, Murnau am Staffelsee, Germany; Medical Park Bernau Chiemsee, Bernau am Chiemsee, Germany

**Keywords:** Proximal humeral fracture, Monoaxial, Polyaxial, Locking plate fixation, Helical blade, Medial support, Calcar

## Abstract

**Background:**

Locking plate fixation of displaced fractures of the proximal humerus is still accompanied by a distinct complication rate, especially in case of osteoporotic bone, short-segment fracture length and comminution of the medial calcar. Secondary loss of reduction leading to varus deformity and screw cutout most frequently lead to surgical revision. The aim of the present study was to evaluate the clinical and radiological outcome of a recently developed polyaxial locking plate that allows for the additional placement of a helical blade device, aiming for support of the medial calcar.

**Methods:**

In this prospective study, 17 patients with a mean age of 63.0 ± 16.0 years suffering from displaced fractures of the proximal humerus (Neer type two-, three- and four-part) were enrolled. All patients were surgically treated using a polyaxial locking plate with additional blade device (group PAB, *n* = 12) or without blade device (group PA, *n* = 5). Functional outcome was recorded using the Munich Shoulder Questionnaire allowing for qualitative self-assessment of the Shoulder Pain and Disability Index (SPADI), the Disability of the Arm, Shoulder and Hand (DASH score) and the Constant Score. Radiological outcome was assessed by analyzing standardized true anterior–posterior and outlet-view radiographs with respect to radiographic evidence of secondary varus displacement, cutout of screws and hardware failure. Results were compared to an age-, gender- and fracture type-matched collective treated by monoaxial locking plate fixation (group MA, *n* = 15).

**Results:**

The mean follow-up was 12.4 ± 2.9 months after surgery. There were no statistical significant differences in clinical outcome in all three groups. Group MA and group PA revealed significant secondary varus displacement in comparison to group PAB at the final follow-up compared to postoperative analysis (*p* < 0.001). The distance between the blade and the articular surface showed no significant increase in group PAB at the final follow-up compared to postoperative analysis. Not-implant-related complications were seen in one and implant-related complications were seen in two patients in group PAB.

**Conclusions:**

Polyaxial locking plate fixation with a blade device to restore medial cortical support reduces the risk of secondary varus displacement even in proximal humeral fractures of the elderly in comparison to monoaxial and polyaxial locking plate fixation without blade insertion.

## Background

Fractures of the proximal humerus account for approximately 5 % of all fractures [1] and occur in a bimodal frequency with younger high-energy and older low-energy mechanisms [2, 3]. Especially in women beyond the age of 40 and in men beyond the age of 60, a significant increase of the rate of proximal humeral fractures is reported [4]. Despite good functional results and satisfactory bony union in radiographs following open reduction and locking plate fixation [5–7], several studies describe frequent complications such as screw cutout or loss of fixation [8–11]. Besides the anatomic reduction, local bone mineral density and age, the restoration of the medial cortical support was identified as a crucial point for preventing secondary failure [6, 12, 13]. Measures to increase the mechanical stability of the medial column of the proximal humerus comprise the achievement of an anatomic or slightly impacted stable reduction as well as the placement of a superiorly directed locking screw in the inferomedial region of the proximal humerus [14].

In this context, the insertion of a helical blade into the femoral head resulted in local bone compaction and significantly improved operative treatment of proximal femur fractures with a lack of medial cortical support [15].

Recently a polyaxial plate with a fixed helical blade for restoration of the medial cortical support has been developed. Therefore, the aim of this prospective study was to evaluate the clinical and radiological outcome of the polyaxial locking plate with an additional blade device after a mean follow-up of 1 year in comparison to a conventional monoaxial locking plate.

## Methods

### Patients

Patients suffering from a displaced fracture of the proximal humerus (displacement >1 cm, angulation of fragments >45-degree angle) presenting to our emergency department were identified and prospectively enrolled. All fractures were classified according to the Neer classification [16]. Preoperative standard radiographs of the proximal humerus (true glenoid anterior–posterior (true a.p.) view and outlet view) and if necessary additional computed tomography was performed to adequately classify the fracture. Patients with open fractures, vascular or neural injury, glenohumeral osteoarthritis (>Samilson II), rotator cuff arthropathy, non-reconstructable bony defects, rotator cuff tears or pathologic fractures were excluded from the study. The decision concerning blade insertion was made based on the intraoperative fracture reduction and the patient’s specific anatomic conditions. In every case, a probe k-wire was drilled over a dedicated k-wire drill guide within the oblong hole. If the anatomic conditions, i.e. small head radius, precluded spiral blade placement a bicortical screw was inserted through the oblong hole into the shaft. Written informed consent was obtained from each patient. The study protocol was approved by the local ethics committee.

An age-, gender- and fracture pattern-matched patient collective was treated by monoaxial locking plate fixation (PHILOS, Synthes^®^) and served as a historical control group (group monoaxial, MA, *n* = 15).

### The implant

**Fig. 1:**
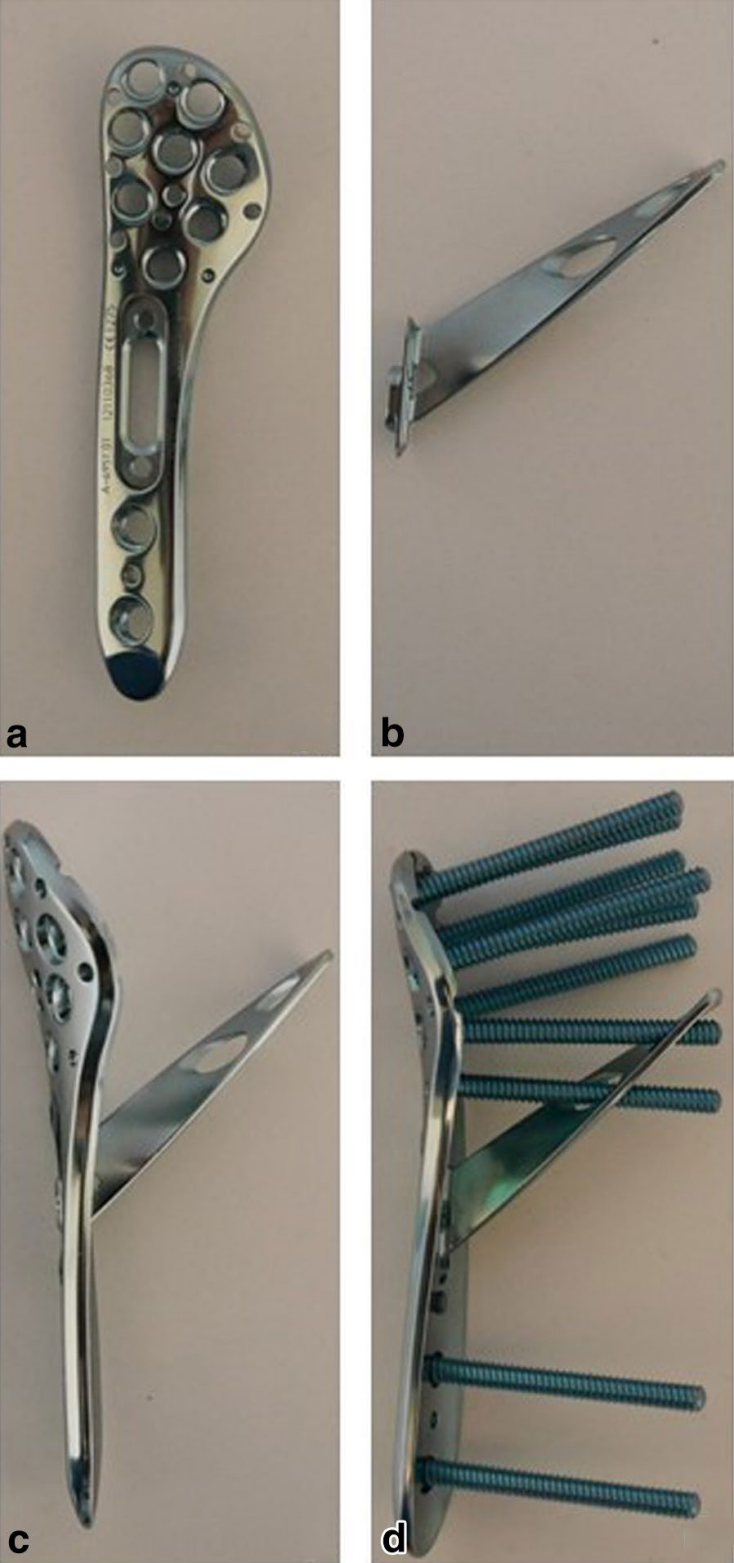
APTUS Proximal Humeral Plate 3.5, Medartis^®^. **a** Plate; **b** helical blade device; **c** plate with helical blade device; **d** completely instrumented plate

The APTUS Proximal Humeral Plate 3.5 (Medartis AG^®^, 4057 Basel, Switzerland) is an anatomically precontoured fixation system with three to seven shaft holes for 3.5 mm locking or 3.5-mm cortex screws and up to eight 3.5-mm locking screws for the humeral head (see Fig. 1). The plate allows for the additional placement of a helical blade, being mounted with two screws to the plate, aiming for a support of the medial calcar.

### Surgical technique and rehabilitation

All patients underwent surgical intervention with open reduction and internal fixation (ORIF), placed in beach chair position with the affected arm in a mobile position. A deltopectoral approach with a curved 12–14-cm-long skin incision between the coracoid process and the proximal humeral shaft was performed. After exposure of the deltopectoral groove and lateralization of the cephalic vein, the deltopectoral groove was dissected and the underlying clavipectoral fascia was incised. The periosteum was sharply dissected and the fracture hematoma was debrided to expose the fracture. Rotator cuff sutures (FiberWire 2, Arthrex^®^, Naples, USA) were placed into the subscapularis, supraspinatus and infraspinatus tendon. K-wires were inserted for temporary fixation of the anatomic reduction whereas the position was checked using fluoroscopy. The plate was centered onto the humeral shaft about 5–8 mm distal to the top of the greater tuberosity. After confirmation of the correct plate positioning in fluoroscopy, screw holes were consecutively drilled. Depending on the intraoperative findings regarding the head radius, the helical blade or an additional bicortical shaft screw was inserted into the oblong blade hole.

On the first postoperative day, the arm was immobilized in a sling and patients started physiotherapy following an active-assisted standard rehabilitation protocol: abduction and flexion were restricted to 90° during the first 6 weeks. With decreasing pain, this training has progressed to strengthening exercises of the rotator cuff and shoulder muscles. Return to sportive activity of the upper extremities was allowed after another 6 weeks.

### Follow-up

**Fig. 2:**
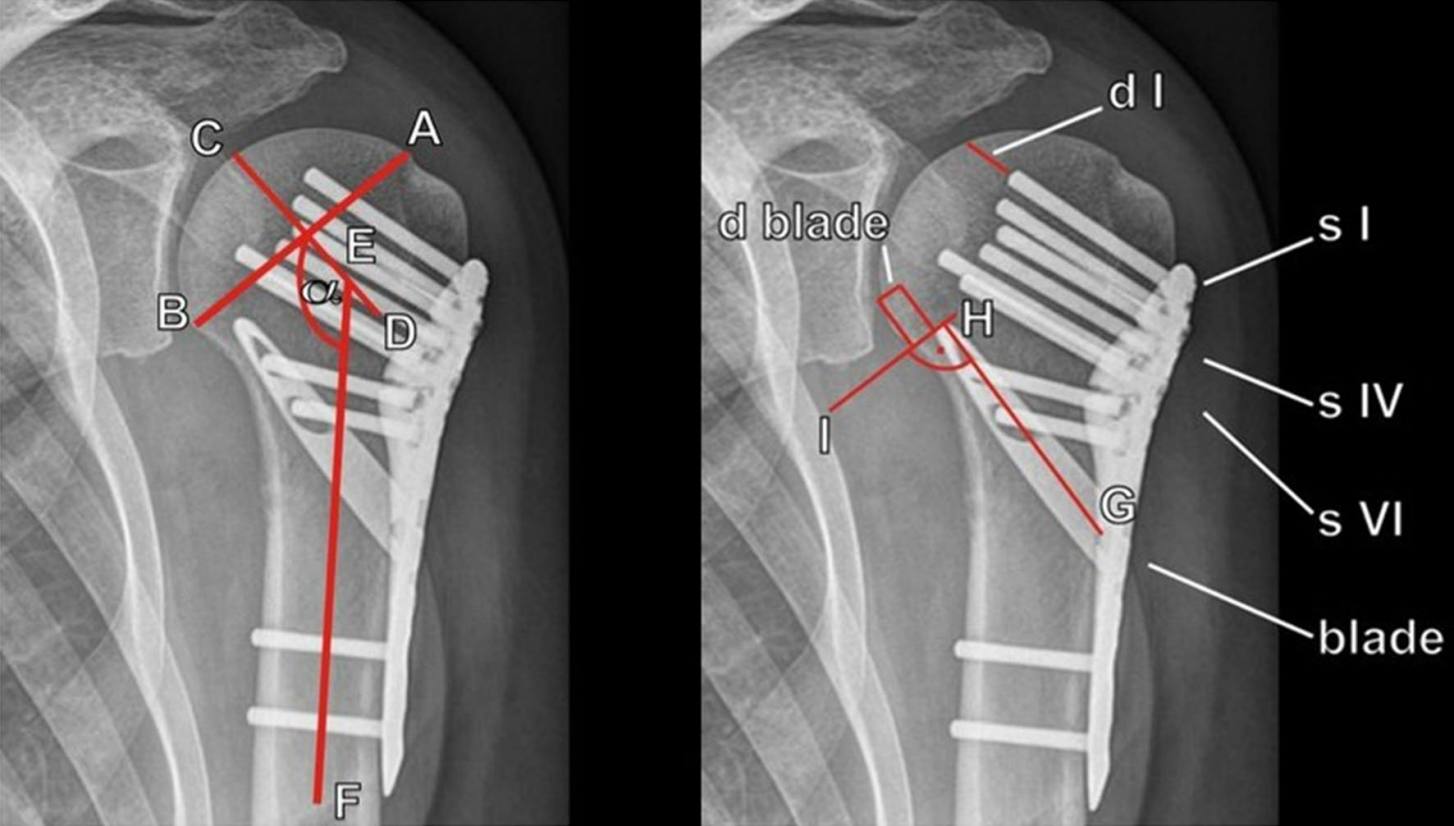
Radiographic evaluation. A line from the superior to the inferior border of the articular surface (*A*–*B* *line*), and then a perpendicular line to the *A*–*B* *line* through the *center* of the humeral head (*C*–*D* *line*) are drawn. The angle (α) between this *line* and the *line* bisecting the humeral shaft (*E*–*F line*) was measured as the head–shaft angle. The distance between the tip of the proximal (s I), the medial (s IV) and the distal (s VI) head screw and the articular surface (d I, IV and VI) was measured. A line bisecting the blade (*G*–*H* *line*) and then a perpendicular line to the *G*–*H* *line* through the tip of the blade were drawn. *D* blade is the distance between the tip of the blade and the articular surface

Clinical and radiological outcomes were assessed in our outpatient clinic during routine postoperative follow-up examinations at 6, 12, 24 and 48 weeks. The Munich Shoulder Questionnaire (MSQ) presents a universally applicable instrument for the self-assessment of shoulder function. It was especially developed for an effective follow-up of shoulder patients allowing for a quantitative assessment of the Shoulder Pain and Disability Index (SPADI), the Disability of the Arm, Shoulder and Hand (DASH score) and the Constant Score. The MSQ has been validated previously and its accuracy and effectiveness for follow-up assessment was sufficiently demonstrated [17–19]. Original Constant Score values were used to calculate a normative age- and sex-specific Constant Score (relative Constant Score) according to Gerber et al. [20]. Radiological assessment contained true a.p. and outlet-view radiographs immediately after surgery as well as 6, 12, 24 and 48 weeks postoperatively to verify fracture alignment and implant position and to identify screw cutout, osteonecrosis, non-union or implant failure. To determine secondary varus dislocation the head–shaft angle was measured drawing a line from the superior to the inferior border of the articular surface (see Fig. 2A–B line) and then a perpendicular line to the A–B line through the center of the humeral head (C–D line). The angle (α) between this line and the line bisecting the humeral shaft (E–F line) was measured as the head–shaft angle [8]. In addition, the distance of the tip of the most cranial head screw (d I), the medial head screw (d IV), the most caudal head screw (d VI) and the blade (d blade) to the articular surface was measured to determine secondary varus dislocation. Two expert shoulder surgeons evaluated all radiographs twice in separate sessions (8 weeks in-between). Consensus decision was made for implant-related failure. Healing was determined by radiographic evidence of bridging bone on true a.p. and outlet-view radiographs.

### Statistics

Data are given in terms of the arithmetic mean ± standard deviation. One-way ANOVA was used to test statistical significance of time of surgery and fluoroscopy. The Kruskal–Wallis one-way analysis of variance on ranks test was used to analyze differences regarding age, sex and fracture pattern. The Friedman repeated measures ANOVA, followed by Holm–Sidak as post hoc was used to detect differences in the head–shaft angle during follow-up. The level of significance was set at *p* < 0.05. Statistical analysis was performed using Sigma Stat 3.1 software (Systat Inc, Chicago, IL, USA).

## Results

### Demographics and fracture morphology

Between July 2012 and July 2013, 17 displaced fractures of the proximal humerus in 17 patients (6 men, 11 women) with a mean age of 63.0 ± 16.0 years were enrolled in the study and surgically treated by polyaxial locking plate fixation in a prospective clinical trial (see Table 1). The mean time interval between trauma and surgery was 2.6 ± 2.1 days. 13 patients suffered from a trauma in terms of fall from a minor height, three patients had a bicycle accident and one patient suffered from falling down the stairs (>three steps). The mean interval between surgery and follow-up was 12.4 ± 2.8 months. According to the Neer classification [16], 3 patients had a two-part, 7 a three-part and 7 a four-part fracture. The historical control group MA consisted of 15 patients (4 men, 11 women) with a mean age of 66.3 ± 13.5 years, a comparable distribution of fracture types (3 two-part, 7 three-part and 5 four-part fractures) and a mean interval between surgery and follow-up of 13.5 ± 2.5 months. There were no statistical significant differences regarding sex, age and fracture pattern.

**Table 1:** Patient demographics and outcomes

No.	Age	Sex	Fracture pattern	Blade	Follow-up (months)	MSQ	SPADI	DASH	Rel. CS	Plate removal	Complications
I	77	F	2-part	N	16	90	98	3	96	N	–
II	74	F	3-part	Y	16	89	92	6	95	N	–
III	65	F	4-part	N	12	87	98	3	85	N	–
IV	75	F	3-part	Y	17	94	94	2	100	N	–
V	70	M	3-part	N	15	81	90	8	71	N	–
VI	54	M	3-part	Y	12	77	81	21	77	Y	Brachial plexus lesion
VII	58	M	2-part	Y	16	91	98	7	93	N	Bent blade
VIII	57	F	3-part	N	14	87	92	8	96	N	–
IX	62	F	4-part	N	11	75	83	26	76	N	–
X	86	F	4-part	Y	11	74	88	20	69	N	–
XI	69	F	4-part	Y	11	69	78	23	61	N	–
XII	69	F	2-part	Y	10	83	88	7	82	N	–
XIII	80	F	4-part	Y	11	77	85	17	77	N	–
XIV	27	M	3-part	Y	11	93	98	2	94	N	–
XV	66	M	4-part	Y	13	69	74	21	54	N	Loss of reduction
XVI	46	F	3-part	Y	7	79	82	17	68	N	–
XVII	36	M	4-part	Y	8	82	82	17	90	N	–

*No* number, *MSQ* Munich Shoulder Questionnaire, *SPADI* Shoulder Pain and Disability Index, *DASH* Disability of the Arm, Shoulder and Hand, *rel. CS* relative Constant Score, *F* female, *M* male, *Y* yes, *N* no

### Surgery characteristics

A 3-hole plate was implanted in 12 patients, whereas a 5-hole plate was used for 5 patients. The helical blade was additionally used in 12 patients. The shaft holes were instrumented so that at least one 3.5-mm locking screw was used. On average 7 holes (6–8 holes) in the humeral head were placed with 3.5-mm locking screws. All procedures were performed by a single surgeon who is an expert in upper extremity surgery. Surgery was performed with an average duration of 93.8 ± 9.2 min in group MA, 95.4 ± 8.5 min in group PA and 101.6 ± 26.3 min in group PAB. The mean dose area product for fluoroscopy accounted for 102.8 ± 51.2 cGycm^2^ in group MA, 103.9 ± 47.9 cGycm^2^ in group PA and 92.2 ± 72.5 cGycm^2^ in group PAB. There were no statistical significant differences regarding duration of surgery and mean area dose of fluoroscopy.

### Complications

**Fig. 3:**
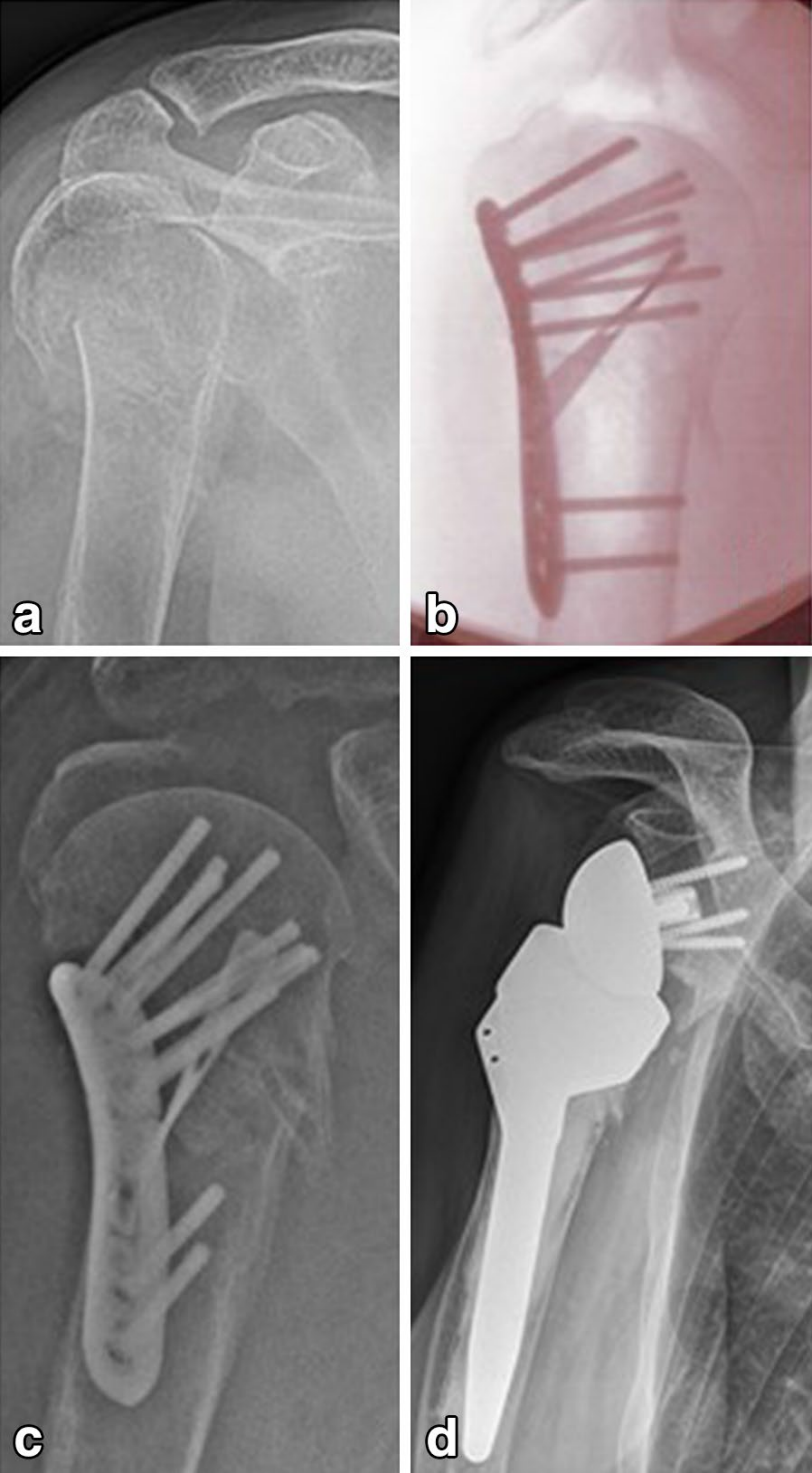
Radiological outcome of a four-part fracture with secondary implantation of a reversed shoulder prosthesis in a 66-year-old man (patient No. XV). **a** Preoperative; **b** intraoperative; **c** postoperative; **d** 13-month follow-up

**Fig. 4:**
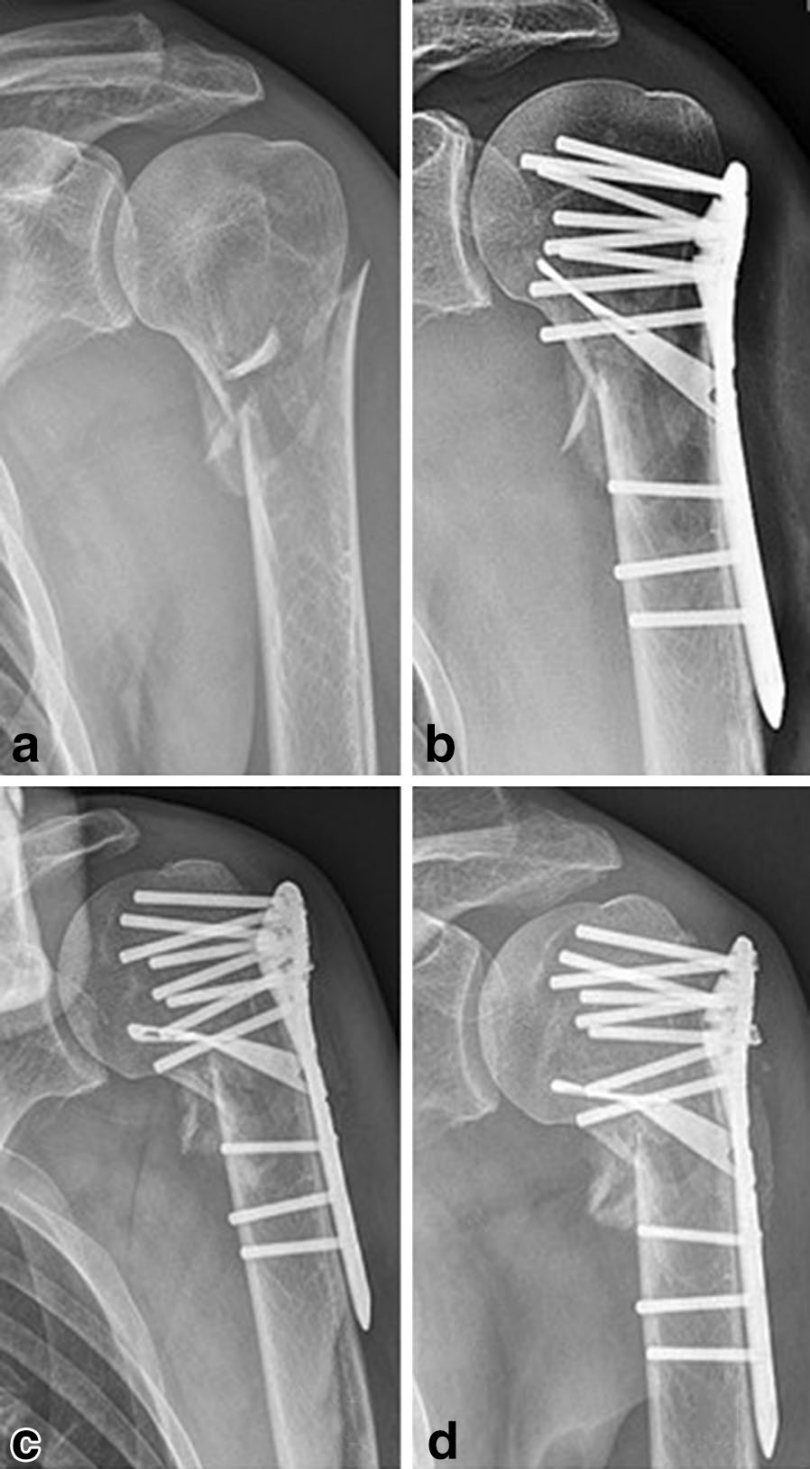
Radiological outcome of a two-part fracture in a 58-year-old man after anew fall on the operated shoulder (patient No. VII). **a** Preoperative; **b** postoperative; **c** 6-week follow-up; **d** 16-month follow-up

Two patients in group PAB presented with implant-associated complications. One patient with a four-part fracture (patient No. XV) demonstrated a subacromial dislocation of the greater tuberosity on the postoperative X-ray control without additional trauma on the second postoperative day (see Fig. 3). Hence, the subsequent removal of the implant and conversion to reversed shoulder arthroplasty was performed. The 2nd patient, who suffered from chronic alcohol abuse (patient No. VII), sustained an anew direct fall on his operated shoulder during the first four postoperative weeks and presented with a distinct loss of reduction. However, after 12 months the fracture has healed in varus deformity (see Fig. 4); though, patient’s self-evaluation resulted in a high patient satisfaction (see Table 1).

In another patient (group PAB, patient No. VI), a not-implant-associated brachial plexus palsy occurred during the first four postoperative weeks. However, neurological symptoms completely recovered until removal of the implant. There were no further complications such as wound-healing problems, infections or implant failures. In 1 of 17 patients, the implant was removed 12 months after surgery as per the patient’s explicit request.

### Patients reported and radiological outcomes

**Fig. 5:**
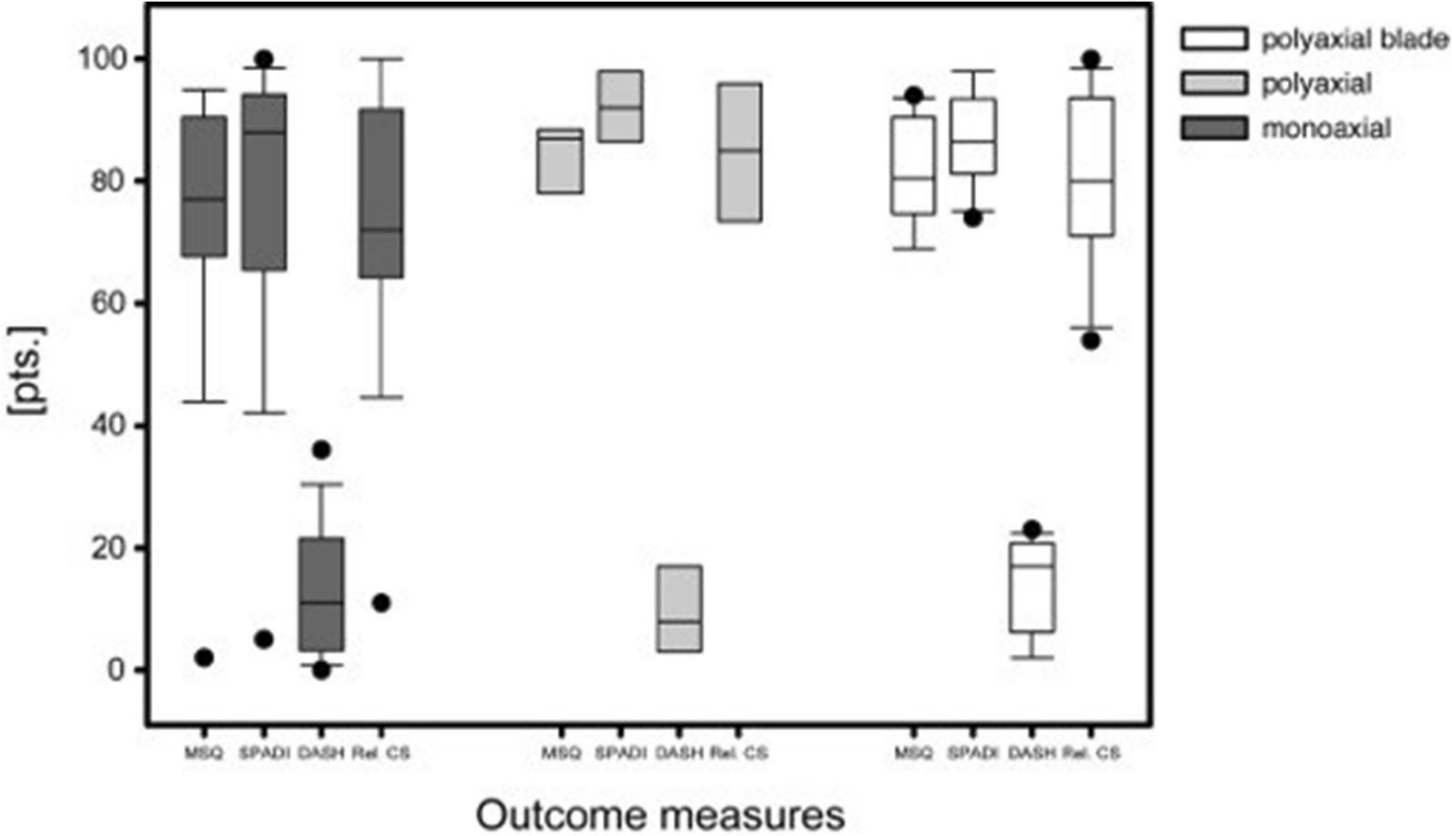
Patient reported outcomes [Munich Shoulder Questionnaire (MSQ), Shoulder Pain and Disability Index (SPADI), Disability of the Arm, Shoulder and Hand (DASH), relative Constant Score (Rel. CS)] of the monoaxial, the polyaxial and the polyaxial + blade group at a mean follow-up of 1 year. Data are given as *vertical box plots* (median *horizontal box line*; 25–75 % interquartile ranges; standard deviations *horizontal line*)

There were no statistical significant differences regarding clinical outcome between the three groups (Table 1; Fig. 5). Bony union occurred in all patients.

**Fig. 6:**
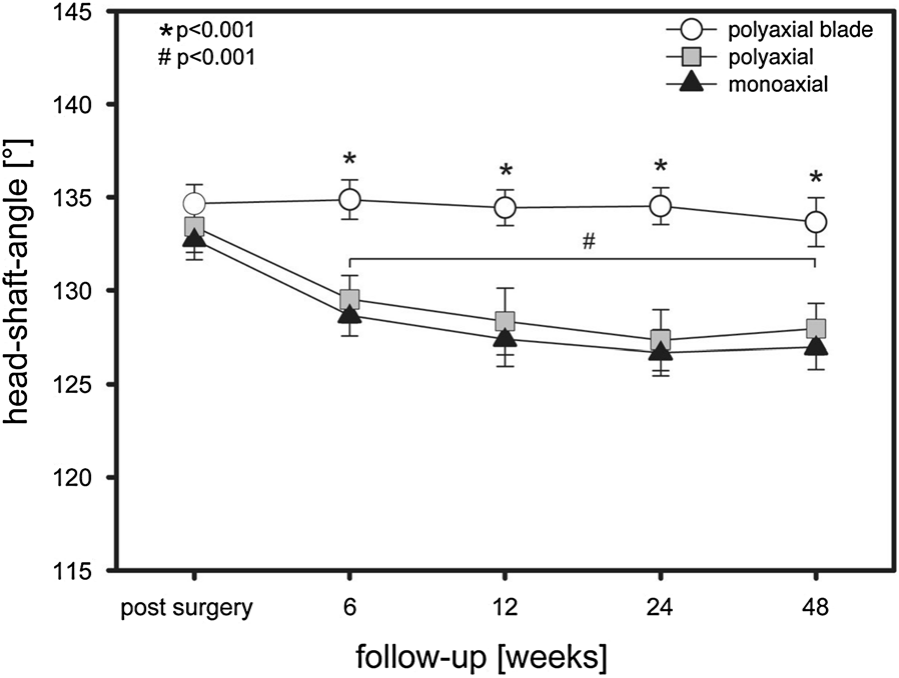
Head–shaft angle. *,^#^ *p* < 0.001

Group MA (*n* = 15) showed a significant secondary varus displacement with a head–shaft angle of 133.1 ± 0.92° at the postoperative analysis compared to the final follow-up examination with 127.0 ± 1.4° (Fig. 6; *p* < 0.001). Group PA (*n* = 5) also showed a significant secondary varus displacement with a head–shaft angle of 134.0 ± 0.87° at the postoperative analysis compared to the final follow-up examination with 127.9 ± 1.25 (Fig. 6; *p* < 0.001). In contrast, group PAB (*n* = 12) showed no statistical significant secondary varus displacement with a head–shaft angle of 134.7 ± 0.96° at the postoperative analysis compared to the final follow-up examination with 133.7 ± 1.26° (Fig. 6).

**Fig. 7:**
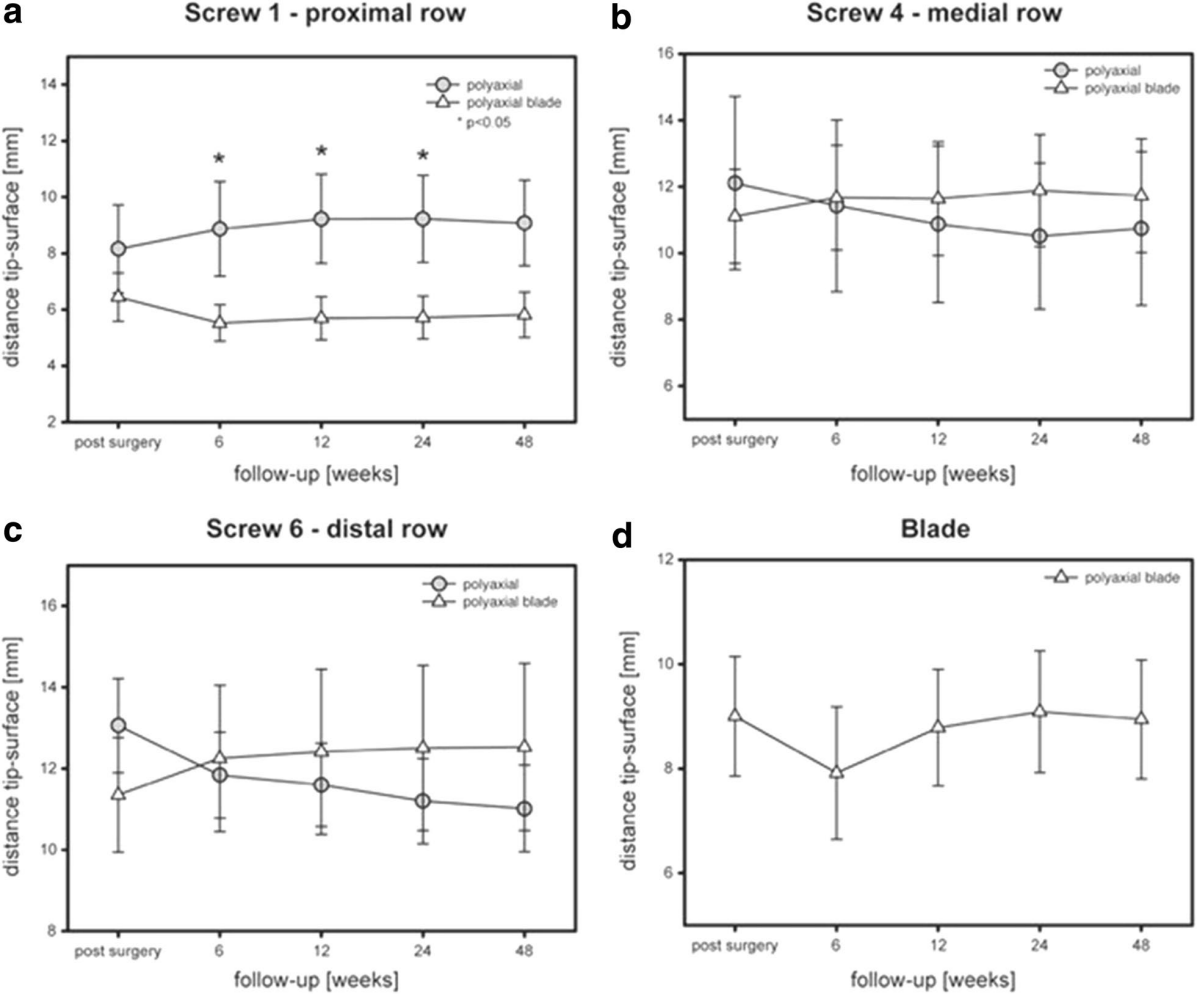
Tip-surface distances. **a** Proximal head screw; **b** medial head screw; **c** distal head screw; **d** blade device. *p < 0.05

In group PA the tip-surface distance of the proximal screw significantly increased whereas the tip-surface distance of the medial and distal screw row decreased during follow-up (Fig. 7). Interestingly, the contrary pattern was observed in group PAB. The tip-surface distance of the proximal screw slightly decreased, whereas the distance of the distal screw increased. However, this motion pattern turned out not to be statistically significant. The distance between the blade and the articular surface showed no significant change comparing the postoperative result and the final follow-up after 12 months (group PAB).

Patient No. II was not available for the 12-week follow-up examination and the patients VIII and XI were not available for the 24-week follow-up examination. All patients underwent the final follow-up examination.

**Fig. 8:**
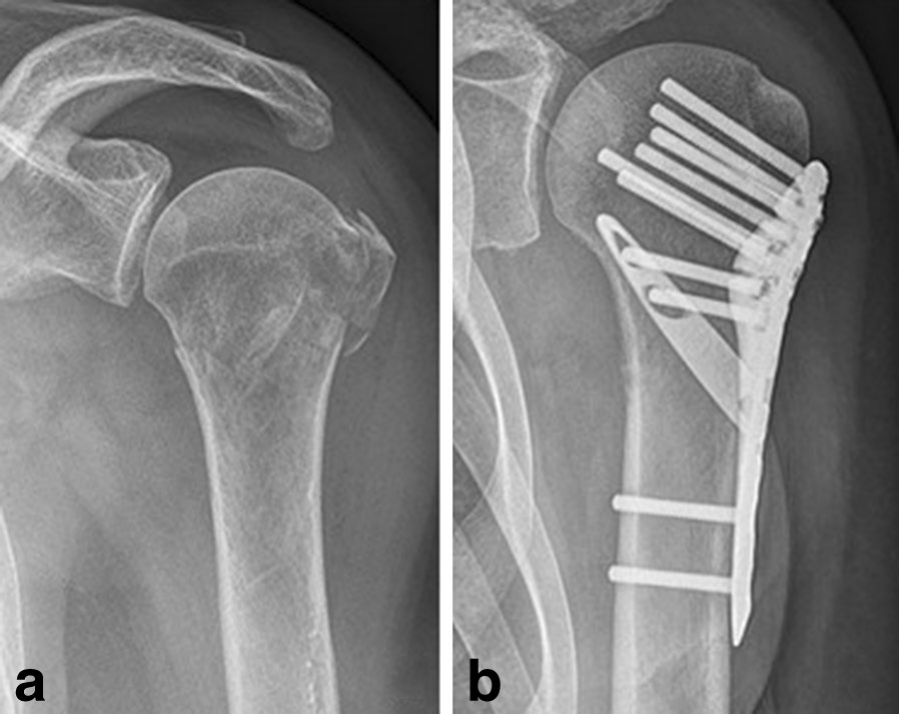
Radiological outcome of a three-part fracture in a 75-year-old woman (patient No. IV). **a** Preoperative; **b** 12-month follow-up

Figure 8 demonstrates the radiological outcome of a three-part fracture of the proximal humerus in a 75-year-old woman at the 12-month follow-up (patient No. IV).

## Discussion

Restoration of the medial cortical support is a crucial point in surgical treatment of proximal humerus fractures to prevent secondary varus collapse of the humeral head. A recently developed locking plate was combined with a helical blade to achieve local bone compaction providing additional bone purchase and an increased stability of the calcar region. In this prospective clinical trial, we compared monoaxial with polyaxial and polyaxial + blade locking plate fixation of proximal humeral fractures. The data of this study demonstrate that all three fixation techniques achieve equally good functional results in patient self-evaluation 1 year after surgery. Radiographic evaluation of standardized true a.p. and outlet-view radiographs postoperatively, 6, 12, 24 and 48 weeks after surgery revealed a statistically significant lower rate of secondary varus displacement in group polyaxial + blade (PAB) in comparison to the monoaxial (MA) and polyaxial (PA) group. Thus, additional blade insertion seems to increase biomechanical stability of the calcar region reducing the varus collapse rate in locking plate fixation of proximal humeral fractures.

The presented study collective consisted of 17 consecutive patients with a mean age of 63.0 ± 16.0 years and a male–female ratio of 6:11 comparable to epidemiologic studies concerning gender distribution and age in proximal humerus fractures [21]. In this clinical trial, we describe not a completely new treatment strategy but a new surgery technique and use of a new implant, respectively, to restore the medial cortical support in proximal humeral fractures. Other authors already described the importance of an intact calcar region [12, 22, 23]. Thus, we consider our results as relevant despite the small number of included patients.

Surgical treatment of proximal humerus fractures is demanding with a partially high implant-related complication rate of up to 44 % such as loss of reduction, screw perforation, impingement or implant failure, leading to a revision rate of up to 30 % in locking plate fixation of three- and four-part fractures [6, 24]. In the presented study, postoperative subacromial dislocation of the greater tuberosity with subsequent conversion of treatment to a reversed shoulder arthroplasty was seen in a displaced four-part fracture of a 66-year-old man. Lenarz et al. [25] reported satisfactory results in primary reverse total shoulder arthroplasty in dislocated four-part fractures in patients older than 65 years. However, in our clinical setting primary osteosynthesis with preservation of the humeral head using plate fixation even in the elderly is preferred due to superior functional results and increased patient satisfaction in comparison to arthroplasty [26, 27].

Neurological complications in proximal humeral fractures such as brachial plexus lesions are extremely rare and mostly associated with anterior fracture dislocation of the shoulder joint [28]. In our study, one patient with a three-part fracture presented with postoperative brachial plexus palsy most likely caused by intraoperative reduction maneuvers. The treatment comprised physical therapy to avoid soft tissue contractures and to strengthen the musculature. Within several months of consequent physical therapy, the patient demonstrated progressive clinical and electrical recovery with diminished signs of myoelectric denervation.

Patient No. XII, a known alcohol addict, presented 4 weeks post-surgery after falling directly onto the operated shoulder alcohol intoxicated in our outpatient clinic. Radiographic control demonstrated a varus dislocation of the humeral head with a bent blade (see Fig. 5). Nevertheless, clinical examination showed a free range of motion and the self-evaluation resulted in a high patient satisfaction (see Table 1). We consider that the additional biomechanical stability due to the inserted blade prevented the humeral head from complete dislocation which enabled bony consolidation in only a mild varus malposition.

Validity of clinical follow-up examinations by the treating and thus operating surgeons themselves is often limited due to observer bias. Furthermore clinical examination not necessarily correlates with subjective impression of the patients in terms of satisfaction. In the presented study, follow-up examination was performed by a self-evaluation questionnaire, the Munich Shoulder Questionnaire, allowing for a qualitative self-assessment of the SPADI, the DASH score and the Constant Score [17, 18]. Good to excellent functional results at a mean follow-up of 1 year after surgery were found, comparable to other authors who used a locking plate for surgical treatment of proximal humeral fractures [22, 29].

In the presented patient cohort, additional blade insertion was not performed in 5 of 17 patients due to a too small head radius. This might cause potential bias. However, from the biomechanical view a small head radius potentially lowers the risk of secondary varus collapse. Therefore, we consider the issue of this specific potential bias as less relevant.

Furthermore, a significant lower rate of secondary varus displacement was found in group PAB in comparison to group MA as well as group PA. Of course radiological outcome does not necessarily correlate with the clinical function. However, Moineau et al. [30] identified varus deformity of the proximal humerus as adverse factor in shoulder arthroplasty which has to be considered in case of posttraumatic glenohumeral osteoarthritis. Zhang et al. [22] reported a reduced failure rate with a medial support screw in locking plate fixation of proximal humeral fractures emphasizing the relevance of an intact calcar region. In comparison to a screw the helical blade used in the described implant of this study achieves superior bone contact surface and the formation of a triangle with two screws locked in the plate (see Fig. 1d) may increase biomechanical stability. Figure 7 shows an increase of d I (Fig. 7a) with a slight decrease of d IV (Fig. 7b) and a crucial decrease of d VI (Fig. 7c) during follow-up examination in group PA which demonstrates the medial head screw as a center of rotation in varus displacement. In group PAB, these distances almost remained constant with a slight increase of the tip-surface distance of the distal screw (Fig. 7c) demonstrating a mild valgus impaction which has been identified as a factor to increase the mechanical stability of the medial column of the humeral head [14]. The tip-surface distance of the blade remained constant during the follow-up examination which is a further fact demonstrating the lack of secondary varus displacement in group PAB.

### Limitations

Several limitations need to be mentioned. The small number of included patients is considered as a limitation. However, since other authors already reported the relevance of an intact calcar region using locking plates in proximal humeral fractures and our study just describes a further option to restore medial cortical support, we consider our results as relevant. A second drawback of our study is of course that the postoperative rehabilitation was done on an outpatient basis and the performance strongly depended on the patient’s compliance despite prescribing physical therapy according to a standard protocol.

## Conclusion

Restoration of the medial cortical support is a crucial point in locking plate fixation of proximal humeral fractures to avoid serious complications such as screw cutout or loss of reduction. All tested locking plates resulted in good functional results with a high patient satisfaction. The additional insertion of an inferomedially placed helical blade significantly reduced the occurrence of secondary varus displacement in comparison to monoaxial and sole polyaxial locking plate fixation. This might lead to a reduction of secondary dislocation in selected patients with comminuted calcar region and inferior bone quality. However, this issue needs to be substantiated by analyzing larger patient cohorts.
